# Pathology of Insanity

**Published:** 1853-07

**Authors:** 


*Pathology of Insanity.*—The following are the conclusions at which Dr. Forbes Winslow has arrived in relation to this vexed subject:

"I believe insanity (I am now referring to persistent insanity, not those transient and evanescent forms of disturbed mind occasionally witnessed) to be the result of a *specific morbid action of the hemispherical ganglia ranging from irritation, passive and active congestion, up to positive unmistakable inflammatory action.* This state of the brain may be confined to one or two of the six layers composing the hemispherical ganglia; but all the layers are generally more or less implicated, in conjunction with the tubular fibres passing from the hemispheres through the vesicular neurine. This specific inflammation, from its incipient to the more advanced stage, is often associated with great vital and nervous depression. It is, like analogous inflammations of other structures, not often accompanied by much constitutional or febrile disturbance, unless it loses its specific features, and approximates in its character to the inflammation of active cerebritis or meningitis. This state of the hemispherical ganglia is frequently conjoined with active sanguineous circulation and congestion, both of the substance of the brain and its investing membranes. The morbid cerebral pathological phenomena—viz: the opacity of the arachnoid, the thickening of the dura mater, its adhesions to the cranium, the depositions so often observed upon the convoluted surface of the hemispheres, and on the meninges, the hypertrophy, scirrhus, the cancerous affections, the induration, the depositions of bony matter in the cerebral vessels and on the dura mater, the serous fluids in, and the ulcerations upon the surface of the ventricles, the alterations in the size, consistence, color, and chemical composition of the vesicular neurine and fibrous portion of the brain—are all, in my opinion, the results, the sequelæ, more or less, of that specific inflammatory condition of the hemispherical ganglia to which I have referred. It does not necessarily follow that the *fons et origo mali* of insanity is invariably to be traced to the brain. The preliminary morbid action and irritation are often situated in the heart, the stomach, the liver, the bowels, the lungs, or the kidneys, the brain being secondarily affected; nevertheless, in all cases inducing actual insanity, the hemispherical ganglia are involved in the morbid action."—*Ibid.*

